# *Helicobacter pylori* CagA and Cag type IV secretion system activity have key roles in triggering gastric transcriptional and proteomic alterations

**DOI:** 10.1128/iai.00595-24

**Published:** 2025-03-06

**Authors:** Jennifer H. B. Shuman, Aung Soe Lin, Mandy D. Westland, Kaeli N. Bryant, Gabrielle E. Fortier, M. Blanca Piazuelo, Michelle L. Reyzer, Audra M. Judd, Tina Tsui, W. Hayes McDonald, Mark S. McClain, Kevin L. Schey, Holly M. Algood, Timothy L. Cover

**Affiliations:** 1 Department of Pathology, Microbiology and Immunology, Vanderbilt University Medical Center 204907 , Nashville, Tennessee, USA; 2 Department of Medicine, Vanderbilt University School of Medicine 5718 , Nashville, Tennessee, USA; 3 Mass Spectrometry Research Center, Vanderbilt University School of Medicine 12327 , Nashville, Tennessee, USA; 4 Department of Biochemistry, Vanderbilt University School of Medicine 12327 , Nashville, Tennessee, USA; 5 Vanderbilt Institute for Infection, Immunology, and Inflammation, Vanderbilt University Medical Center 12328 , Nashville, Tennessee, USA; 6 Veterans Affairs Tennessee Valley Healthcare System, Nashville, Tennessee, USA; Stanford University School of Medicine, Stanford, California, USA

**Keywords:** *Helicobacter pylori*, gastric cancer, inflammation, carcinogenesis, microbiome, biomarkers, transcriptomics, proteomics

## Abstract

Colonization of the human stomach with *cag* pathogenicity island (PAI)-positive *Helicobacter pylori* strains is associated with increased gastric cancer risk compared to colonization with *cag* PAI-negative strains. To evaluate the contributions of the Cag type IV secretion system (T4SS) and CagA (a secreted bacterial oncoprotein) to gastric molecular alterations relevant for carcinogenesis, we infected Mongolian gerbils with a Cag T4SS-positive wild-type (WT) *H. pylori* strain, one of two Cag T4SS mutant strains (∆*cagT* or ∆*cagY*), or a ∆*cagA* mutant for 12 weeks. Histologic staining revealed a biphasic distribution of gastric inflammation severity in WT-infected animals and minimal inflammation in animals infected with mutant strains. Atrophic gastritis (a premalignant condition), dysplasia, and gastric adenocarcinoma were only detected in WT-infected animals with high inflammation scores. Transcriptional profiling, liquid chromatography-tandem mass spectrometry analysis of micro-extracted tryptic peptides, and imaging mass spectrometry revealed more than a thousand molecular alterations in gastric tissues from WT-infected animals with high inflammation scores compared to uninfected tissues and few alterations in tissues from other groups of infected animals. Proteins with altered abundance in animals with severe Cag T4SS-induced inflammation mapped to multiple pathways, including the complement/coagulation cascade and proteasome pathway. Proteins exhibiting markedly increased abundance in tissues from *H. pylori*-infected animals with severe inflammation included calprotectin components, proteins involved in proteasome activation, polymeric immunoglobulin receptor (PIGR), interferon-inducible guanylate-binding protein (GBP2), lactoferrin, lysozyme, superoxide dismutase, and eosinophil peroxidase. These results demonstrate key roles for CagA and Cag T4SS activity in promoting gastric mucosal inflammation, transcriptional alterations, and proteomic alterations relevant to gastric carcinogenesis.

**IMPORTANCE:**

*Helicobacter pylori* colonizes the stomachs of about half of humans worldwide, and its presence is the primary risk factor for the development of stomach cancer. *H. pylori* strains isolated from humans can be broadly classified into two groups based on whether they contain a chromosomal *cag* pathogenicity island, which encodes a secreted effector protein (CagA) and components of a type IV secretion system (T4SS). In experiments using a Mongolian gerbil model, we found that severe gastric inflammation and gastric transcriptional and proteomic alterations related to gastric cancer development were detected only in animals infected with a wild-type *H. pylori* strain containing CagA and an intact Cag T4SS*.* Mutant strains lacking CagA or Cag T4SS activity successfully colonized the stomach without inducing detectable pathologic host responses. These findings illustrate two different patterns of *H. pylori*-host interaction.

## INTRODUCTION

Approximately half of the human population are colonized with the bacterium *Helicobacter pylori* (1). Though most of these infections are asymptomatic, *H. pylori* infection can lead to gastric or duodenal ulcers, gastric adenocarcinoma, or gastric lymphoma (2, 3). Colonization by *H. pylori* is the primary risk factor for the development of gastric adenocarcinoma, which is a leading cause of cancer-related mortality globally (2–5). Therefore, *H. pylori* has been classified as a Group 1 carcinogen (6).

Within the stomach, *H. pylori* can colonize multiple niches. For example, *H. pylori* can colonize two different regions of the stomach (corpus and antrum) (7–9). The corpus, also known as the body of the stomach, contains specialized epithelial cells that secrete stomach acid (parietal cells) and digestive enzymes (chief cells) (10). The antrum contains specialized G and D cells that influence gastric acid secretion by parietal cells (11). Within both the antrum and the corpus, *H. pylori* populations can be detected in both the superficial gastric mucus and within gastric glands (12).

The outcome of *H. pylori* infection is influenced by many factors, including *H. pylori* genetic variation, host genetic variation, and diet (3, 13–15). One of the *H. pylori* strain-specific features widely recognized as a determinant of clinical outcome is a ~40 kb chromosomal region known as the *cag* pathogenicity island (PAI) (16, 17). The *cag* PAI is present in about 50%–60% of clinical isolates from the United States. The proportion of clinical isolates containing the *cag* PAI is substantially higher in East Asia and several other parts of the world, compared to the United States (17). Epidemiologic studies have shown that gastric colonization with *cag* PAI-positive strains of *H. pylori* is associated with increased gastric cancer risk compared to colonization with *cag* PAI-negative strains (17). The *cag* PAI encodes components of the Cag type IV secretion system (T4SS) and the secreted effector protein CagA, which has been designated as a bacterial oncoprotein (18, 19). The Cag T4SS delivers CagA, lipopolysaccharide (LPS) metabolites (such as ADP-heptose), peptidoglycan, and bacterial DNA into host cells. CagA interacts with multiple host cell proteins, leading to changes in cell signaling (20–26). CagA null mutant strains retain the ability to activate NF-kB and stimulate IL-8 production in gastric epithelial cells (24, 27), which is attributable to Cag T4SS-dependent delivery of LPS metabolites or peptidoglycan into host cells (20–26, 28).

A stepwise progression of gastric pathologic alterations, known as the “Correa Cascade” (15, 29), begins with non-atrophic gastritis (gastric mucosal inflammation in response to *H. pylori*), which can progress to multifocal atrophic gastritis (29). Atrophic gastritis can then progress to intestinal metaplasia, followed by dysplasia (30). Once atrophic gastritis and dysplasia develop, the risk of progression to adenocarcinoma is almost 10-fold higher than in individuals who have non-atrophic gastritis (31, 32). Evaluation of hematoxylin and eosin (H&E)-stained gastric tissue has been the standard in the field for detecting these pathological outcomes of infection.

Our understanding of the pathogenesis of *H. pylori* infection is aided by experimentation with animal models. Rodent stomachs contain glandular antrum and corpus regions, as do human stomachs (33). Both mouse and Mongolian gerbil models are commonly used to investigate *H. pylori* colonization, gastric pathology, and effects of *H. pylori* virulence factors (34–36). While *H. pylori* can experimentally colonize the stomach in both mice and Mongolian gerbils, there are notable differences in the gastric response to infection. Both species can develop gastric mucosal inflammation, but the immune response tends to be more severe in Mongolian gerbils than in wild-type mice (35, 36). Wild-type mice rarely develop premalignant or malignant disease in response to *H. pylori* infection, whereas Mongolian gerbils can develop premalignant and malignant gastric lesions (8, 35–47). Multiple studies have shown that CagA and Cag T4SS activity contribute to the development of gastric disease in the Mongolian gerbil model (8, 37, 40, 41, 43–46). Specifically, a CagA-positive *H. pylori* strain with intact Cag T4SS activity can cause severe gastric inflammation, gastric ulceration, and/or gastric cancer in the Mongolian gerbil model, whereas CagA mutant strains or mutant strains lacking Cag T4SS activity cause less severe gastric inflammation and typically do not cause gastric ulceration or cancer in this model (8, 37, 40, 41, 43–46). Thus far, there has been relatively little effort to define *H. pylori*-induced gastric molecular alterations in the Mongolian gerbil model (47–49).

The goal of the current study was to evaluate the role of CagA and Cag T4SS in *H. pylori*-induced gastric molecular alterations. To accomplish this goal, we used transcriptional profiling, liquid chromatography-tandem mass spectrometry (LC-MS/MS), and imaging mass spectrometry to analyze the gastric immune responses and gastric molecular alterations that occur in Mongolian gerbils infected with a wild-type (WT) *H. pylori* strain compared to immune responses and molecular alterations that occur in animals infected with mutant strains lacking CagA or Cag T4SS activity. Consistent with previous studies (8, 35–44, 46, 47), we report that an *H. pylori* WT strain has the potential to incite severe gastric inflammation, but there is a biphasic distribution in the severity of the gastric mucosal inflammatory response in gerbils infected with the WT strain. We report that CagA-mutant and Cag T4SS-mutant strains cause minimal detectable transcriptional or proteomic alterations. We also show that WT-infected animals with low levels of gastric inflammation have very few detectable gastric molecular alterations and thus resemble animals infected with CagA or T4SS mutant strains. These results reveal key roles of CagA and the Cag T4SS in triggering gastric mucosal inflammation and transcriptional and proteomic remodeling of the stomach.

## RESULTS

### A WT *H. pylori* strain, but not Cag T4SS- or CagA-deficient mutants, can induce severe gastric inflammation, atrophic gastritis, and gastric cancer

Three mutant strains (*ΔcagA*, *ΔcagT*, and *ΔcagY*) were derived from *H. pylori* strain 7.13 (a WT Cag T4SS-positive and CagA-positive strain), as described in Materials and Methods. Consistent with previous results (27, 46, 50), the *cagT* and *cagY* mutants lacked Cag T4SS activity, whereas a Δ*cagA* mutant retained Cag T4SS activity (Fig. S1). Male and female Mongolian gerbils were infected with each of these strains, and control animals received bisulfite-free Brucella broth alone. After 12 weeks, animals were euthanized, and the stomachs were processed to allow subsequent analyses. Analyses of gastric tissues from subsets of animals in the uninfected, WT-infected, or ∆*cagT* mutant-infected groups were reported in a previous study (49); the current study reports new analyses of the tissues from all five groups of animals, including analyses of additional gastric tissues in each group (uninfected, infected with the WT strain, and infected with ∆*cagT*, ∆*cagY*, or ∆*cagA* mutants).

**Fig 1:**
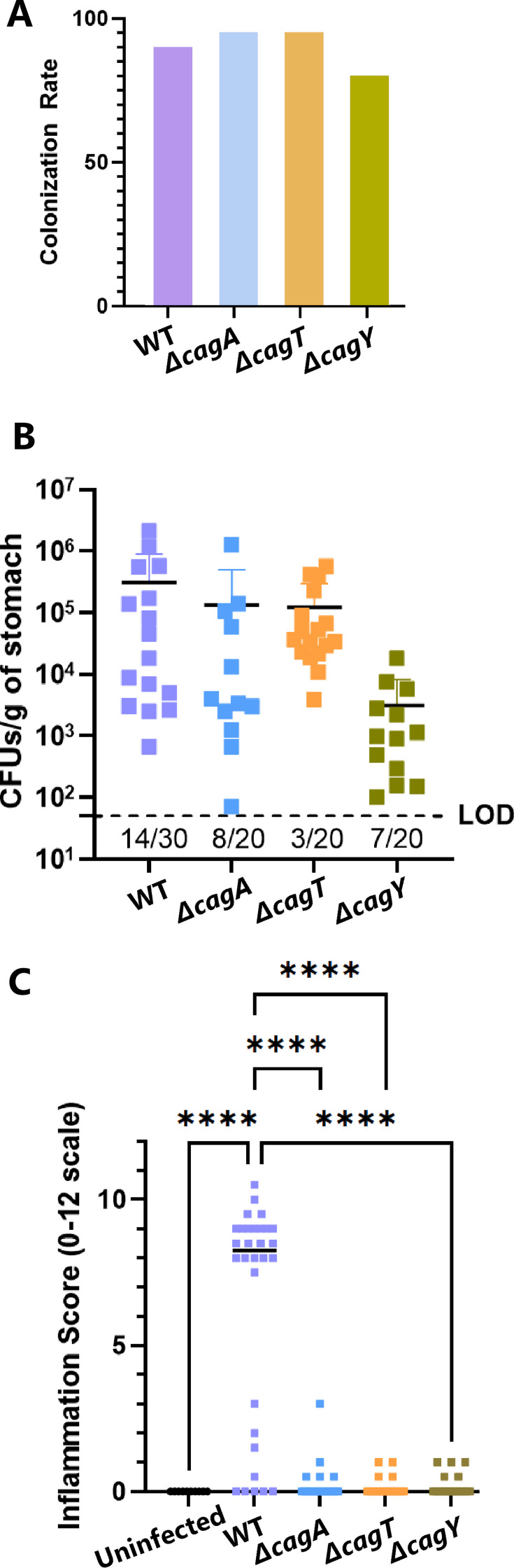
Colonization of Mongolian gerbil stomachs by WT and mutant *H. pylori* strains. (**A**) Colonization rates for animals infected with the WT strain (*n* = 30) or mutant strains (*ΔcagA*, *ΔcagT*, or *ΔcagY*, each *n* = 20), based on the successful culture of *H. pylori* or the presence of *H. pylori* detected by Steiner staining of gastric tissues at the endpoint of infection. The colonization rate refers to the number of animals with positive cultures and/or positive Steiner stains divided by the total number of experimentally infected animals, expressed as a percentage. There was no statistical difference in colonization rates among groups (one-way ANOVA). (**B**) *H. pylori* colonization density of infected animals. Each point represents results for a single animal. The numbers below the limit of detection (LOD) for each group represent the numbers of animals in that group from which *H. pylori* was not successfully cultured. There was no statistical difference in bacterial density among groups (one-way ANOVA analysis of culture-positive animals). (**C**) Overall gastric inflammation scores for all animals, including those with negative cultures and negative Steiner stains. The inflammation score is on a 0–12 scale, based on analysis of acute and chronic inflammation in the antrum and corpus. Asterisks indicate *P* < 0.0001 (one-way ANOVA with Benjamini-Hochberg FDR correction).

The proportion of experimentally infected animals from which *H. pylori* was successfully cultured or detected by Steiner staining was similar in animals infected with the WT strain compared to animals infected with mutant strains, ranging from 80% to 95% (Fig. 1A). The *H. pylori* colonization densities in animals infected with the *cag* mutant strains were similar to those in animals infected with the WT strain (Fig. 1B).

To compare the gastric inflammatory responses to WT or mutant *H. pylori* strains, H&E-stained tissues were histologically evaluated. Acute inflammation (neutrophils) and chronic inflammation (mononuclear cells) in the gastric corpus and antrum were scored on scales of 0–3, leading to an overall inflammation score of 0–12 for each animal (Fig. 1C). Inflammation was absent in all uninfected animals, and inflammation scores ranged from 0 to 10.5 in infected animals (Fig. 1C). Only Mongolian gerbils infected with the WT strain exhibited cumulative inflammation scores greater than 3. Animals experimentally infected with the WT *H. pylori* strain exhibited inflammation scores in a biphasic distribution, with about 30% of the WT-infected animals exhibiting scores of 3 or lower (the “low inflammation” group) and the remaining 70% of WT-infected animals exhibiting scores of >7 (the “high inflammation” group). In comparison, only 20%–25% of animals infected with the Cag T4SS or CagA mutants exhibited detectable inflammation, and all mutant-infected animals had inflammation scores of 3 or lower (similar to the “low inflammation” group of WT-infected animals; Fig. 1C). In these animals infected with the *cag* mutants, inflammation was limited to the antrum and absent from the corpus; the antral inflammation caused by *cag* mutants was classified as chronic (mononuclear cell) inflammation, with minimal detectable acute (neutrophil) inflammation.

Severe gastric disease states were detected in a subset of animals infected with the WT strain but not in uninfected or mutant-infected Mongolian gerbils. Only WT-infected animals with inflammation scores above 7 had evidence of gastric ulcers (restricted to the antrum), corpus atrophy, low- or high-grade dysplasia, or invasive adenocarcinoma. In some cases, individual animals exhibited multiple gastric disease states. All 21 WT-infected animals with high inflammation scores exhibited detectable corpus gland atrophy (ranging from approximately 33% to 67% loss of parietal cells) and at least low-grade dysplasia in the antrum. Nine (43%) of these animals exhibited some level of corpus dysplasia, and seven of the nine had invasive adenocarcinoma. Overall, animals infected with the WT strain had varied outcomes (ranging from no detectable gastric disease to severe disease), while none of the animals infected with Cag T4SS or CagA mutant strains had detectable gastric disease (except for mild gastritis in some animals).

**Fig 2:**
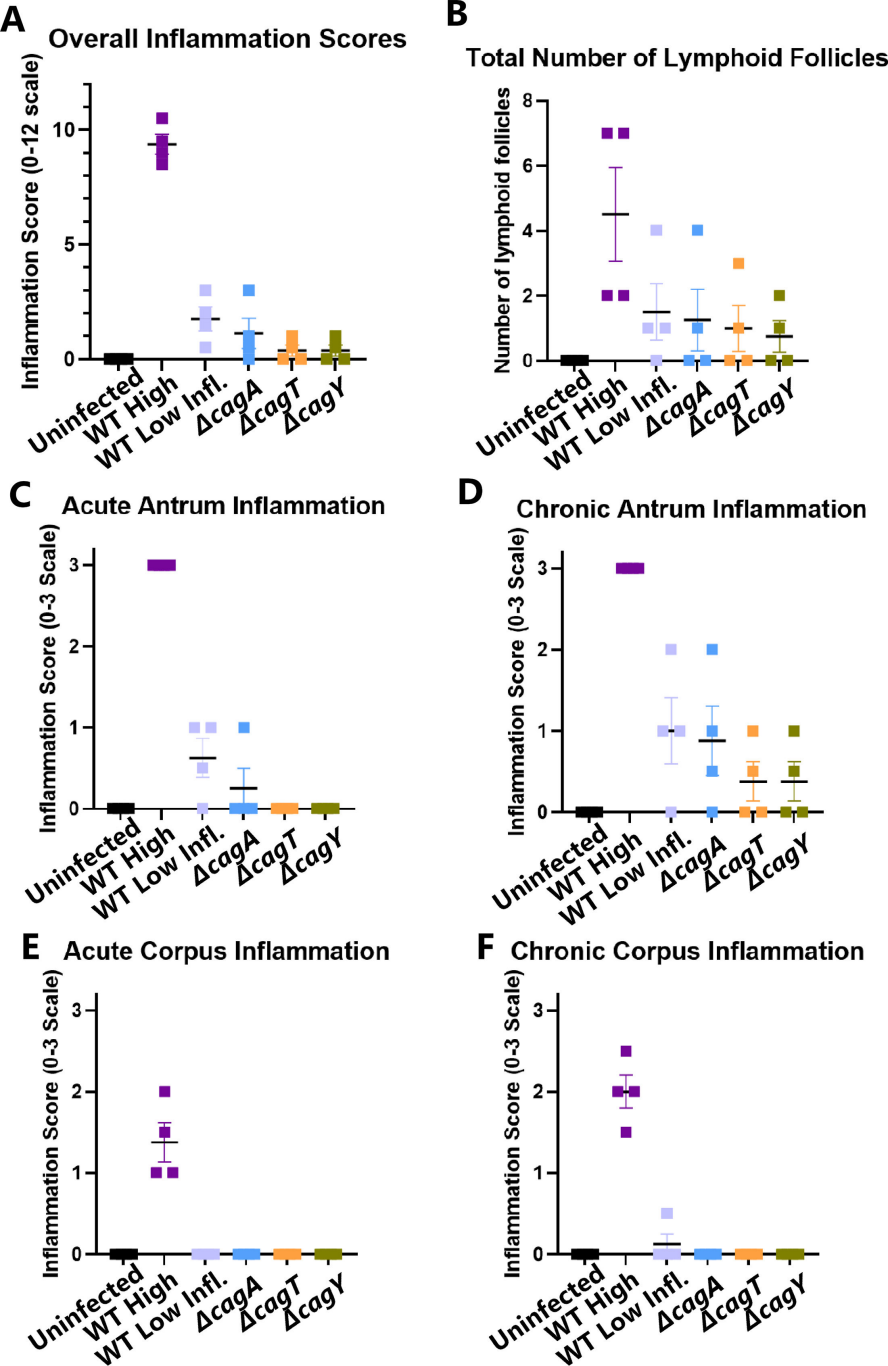
Gastric inflammation in the subset of Mongolian gerbils selected for transcriptional profiling. (**A**) Overall inflammation score on a 0–12 scale, based on analysis of acute and chronic inflammation in the antrum and corpus. (**B**) Total number of lymphoid follicles visible in each tissue section, including both antrum and corpus. (**C** and D) Acute and chronic inflammation in the antrum. (**E**) and (F) acute and chronic inflammation in the corpus.

For further analyses (described below), we selected 4–5 stomach samples from six groups of Mongolian gerbils; these included uninfected gerbils, gerbils infected with the WT strain and exhibiting high levels of gastric inflammation (total inflammation score >7), gerbils infected with the WT strain and exhibiting low levels of gastric inflammation (total inflammation score <3), and animals infected with mutant strains (*ΔcagA*, *ΔcagT*, or *ΔcagY* mutants). The inflammatory profiles of these representative animals are shown in Fig. 2.

### CagA and Cag T4SS activity drive the host transcriptional response to infection

**Fig 3:**
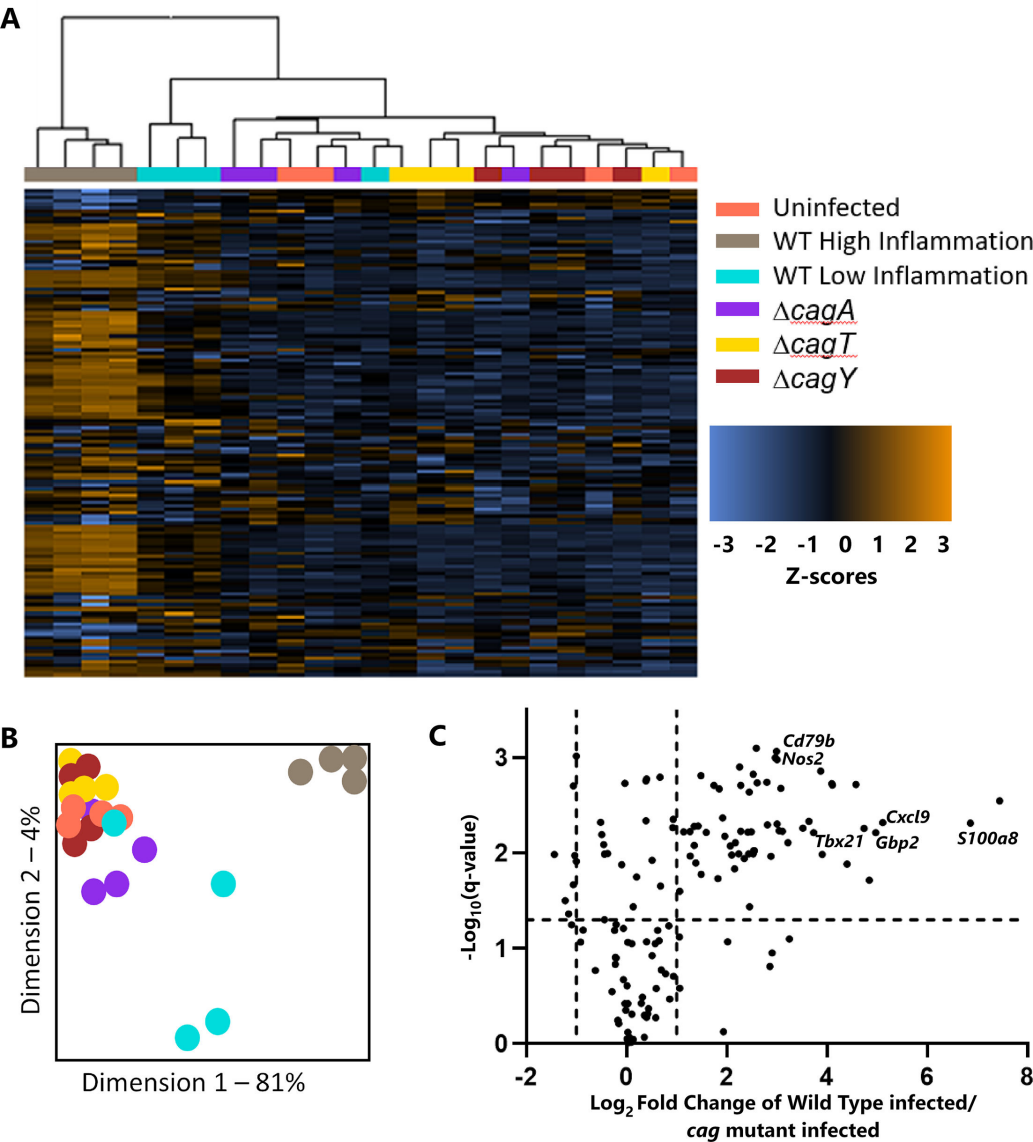
Gastric transcriptional profiles of Mongolian gerbils. Pink labels denote uninfected gerbils, gray labels denote WT-infected gerbils with high inflammation (inflammation scores >7), teal labels denote WT-infected animals with low inflammation (inflammation scores <3), purple labels denote animals infected with a *ΔcagA* mutant, yellow labels denote animals infected with a *ΔcagT* mutant, and red labels denote animals infected with a *ΔcagY* mutant. (**A**) Heat map of transcriptional profiles generated using unsupervised agglomerative clustering. (**B**) Principal component analysis of results. (**C**) Volcano plot comparing the transcriptional profiles of WT-infected animals (including both the high and low inflammation groups) to the profiles of all *cag* mutant-infected animals. The horizontal dashed line indicates a *P*-value of 0.05, (Benjamini-Hochberg multiple test correction). The vertical dashed lines indicate a fold change of −2 or 2. Representative genes exhibiting marked differences in transcript levels include immunoglobulin-associated beta (*Cd79b*), nitric oxide synthase-2 (*Nos2*), T box transcription factor 21 (*Tbx21*), CXC motif chemokine ligand 9 (*Cxcl9*), guanylate-binding protein 2 (*Gbp2*), and a calprotectin component (*S100a8*). Complete data are shown in Table S1.

To detect gastric transcriptional alterations linked to CagA or Cag T4SS activity, we used a multiplex nucleic acid hybridization assay designed to interrogate the gastric mucosal inflammatory response. RNA was isolated from whole stomach strips (including both the corpus and antrum), and transcriptional profiles were quantified using a NanoString panel designed for the Mongolian gerbil model (49). Heat map, dendrogram, and principal component analyses showed that the transcriptional profiles of animals in the WT- infected group (and especially those in the WT-infected high inflammation group, denoted by gray symbols) were markedly different from those of animals in the uninfected and mutant-infected groups (Fig. 3A and B).

To identify differences in transcript levels of specific genes, we compared the gastric transcriptional profiles of WT-infected animals (both the high- and low-inflammation groups) with the gastric transcriptional profiles of *cag* mutant-infected animals (including animals infected with Δ*cagA*, Δ*cagT*, or Δ*cagY* mutants; Fig. 3C). The transcript abundances of 89 of the 149 genes on the panel were significantly different in this comparison (Table S1). Most of the genes exhibiting increased transcript abundance in the WT-infected animals reflect the presence of a gastric mucosal inflammatory response. Markers of an upregulated adaptive immune response included transcripts associated with B cells (*Cd79b* and *Ms4a1*) and T cells (*Tbx21*, indicating the presence of a Th1 response). Other upregulated transcripts include genes encoding neutrophil components (*S100a8* and *S100a9* subunits of calprotectin) and genes involved in producing reactive nitrogen species (*Nos2*). Transcripts encoding proinflammatory chemokines, including *Cxcl9*, *Il1b*, *Cxcl1,* and *Ccl8*, were also increased in abundance. Collectively, these results indicated that WT-infected animals had a gastric transcriptional profile characterized by a more intense gastric inflammatory response to infection than the transcriptional profile of mutant-infected animals, consistent with the histologic analysis.

**Fig 4:**
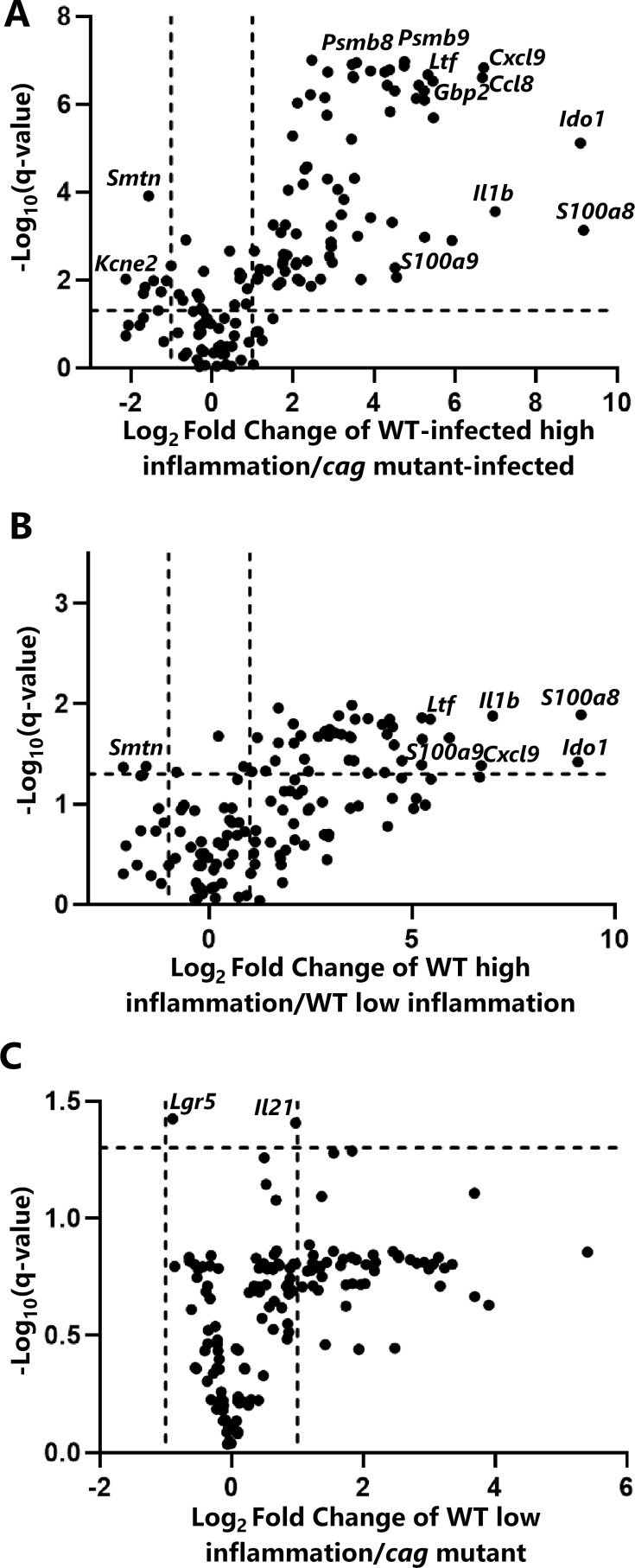
Gastric transcriptional alterations in animals infected with the WT strain correlate with high inflammation scores. The horizontal dashed line indicates a *P*-value of 0.05. The vertical dashed lines indicate fold changes of −2 or 2. (**A**) Volcano plot comparing the transcriptional profiles of WT-infected animals with high inflammation (scores >7) to those of *cag* mutant-infected animals. (**B**) Volcano plot comparing the transcriptional profiles of WT-infected animals with high inflammation scores (scores >7) to those of WT-infected animals with low inflammation scores (scores <3). (**C**) Volcano plot comparing the transcriptional profiles of WT-infected animals with low inflammation to the *cag* mutant-infected animals. Genes exhibiting marked differences in transcript levels (panels A and B) include a calprotectin component (*S100a8*), interleukin-1 beta (*Il1b*), indoleamine 2,3-dioxygenase 1 (*Ido1*), potassium voltage-gated channel subfamily E regulatory subunit 2 (*Kcne2*), and smoothelin (*Smtn*). Complete data are shown in Table S3 and S4.

We conducted additional analyses to differentiate WT-infected animals exhibiting high levels of gastric inflammation (inflammation score >7) and WT-infected animals exhibiting lower levels of gastric inflammation (inflammation score <3). Comparison of the WT-infected “high inflammation” group to the *cag* mutant-infected group revealed 100 significant differences in transcript levels (Fig. 4A; Table S2). The transcripts with the highest fold-changes and most significant differences when comparing the WT-infected animals with high inflammation to the *cag* mutant-infected group included those encoding calprotectin subunits *S100a8* and *S100a9*, proteasome subunits *Psmb8* and *Psmb9*, lactoferrin (*Ltf*), interleukin-1 beta (*Il1b*), the immune response regulator *Ido1*, the chemotaxis-inducer *Cxcl9*, *Gbp2* (an interferon-inducible GTPase) (51), and the parietal cell component *Kcne2*. When the WT high-inflammation group was compared to the WT low-inflammation group (Fig. 4B; Table S3), transcripts for 48 genes exhibited significantly different abundance, including an increased abundance of dozens of proinflammatory genes, similar to those identified when comparing the WT-infected group to the mutant-infected group. The similar results when comparing the WT-infected with high inflammation group to either the WT-infected low inflammation group or the *cag* mutant-infected group suggest that the transcriptional differences between WT- and mutant-infected animals (Fig. 3C) are driven almost entirely by the robust inflammatory response to infection, which is dependent on CagA and a functional Cag T4SS.

When comparing the WT-infected low-inflammation group to the *cag* mutant-infected group, only two statistically significant differences were identified: *Lgr5*, a marker of gastric epithelial stem cells (52), and *Il21*, a cytokine with roles in promoting Th1 and Th17 inflammation (53, 54). Transcript levels of these two genes were increased in gastric tissues from animals in the WT low-inflammation group compared to tissues from the *cag* mutant-infected group (Fig. 4C and Table S4).

When the gastric transcriptional profiles of animals infected with *cag* mutant strains were compared to those of uninfected animals, there were no significant differences. In contrast to our expectations, there were no differences when comparing the transcriptional profiles of *ΔcagA* mutant-infected stomachs to those of *ΔcagT-* and *ΔcagY*-infected stomachs.

### LC-MS/MS analysis reveals gastric proteomic alterations dependent on the Cag T4SS and CagA

Next, we examined Cag T4SS- and CagA-dependent and -independent effects of *H. pylori* infection on the host proteome using a quantitative LC-MS/MS proteomics method. This approach allowed an untargeted assessment of a much larger number of proteins than the relatively small number of transcripts evaluated with the NanoString panel. Tryptic peptide digestions were performed separately for the corpus and antrum regions of each stomach (using strips of stomach tissue from the same animals analyzed by NanoString methods). The resulting micro-extracted samples were analyzed by LC-MS/MS, which allowed for quantification of the abundance of thousands of peptides (and corresponding proteins).

We analyzed LC-MS/MS data from five groups of animals (uninfected or infected with WT, *ΔcagA*, *ΔcagT*, or *ΔcagY* strains). To begin our analysis with the simplest approach possible, we undertook pairwise comparisons of results for the same gastric regions (corpus or antrum) among all groups of animals (uninfected or infected with the WT, *ΔcagA*, *ΔcagT*, or *ΔcagY* strains). When comparing corpora or antra from the WT-infected group with high inflammation to corpora or antra from other groups, there were hundreds of statistically significant differences in protein abundance. However, pairwise comparisons among the other groups did not yield statistically significant differences. Specifically, we did not detect significant differences when comparing tissues from any of the mutant-infected groups to tissues from the uninfected group. In other words, we did not detect any proteomic signatures of *H. pylori* infection in animals persistently colonized with Cag T4SS or CagA mutants. In addition, no significant differences were detected when comparing tissues from CagA mutant-infected animals to tissues from Cag T4SS mutant-infected animals.

To learn more about how the Cag T4SS and CagA may affect the gastric proteome, we compared the LC-MS/MS results of corpora (Table S5) or antra (Table S6) from WT-infected animals to LC-MS/MS results of corresponding gastric regions from *ΔcagA*, *ΔcagT*, and *ΔcagY-*infected animals grouped together. This comparison revealed 1,312 significant differences (including both the corpora-to-corpora and the antra-to-antra comparisons). One hundred eighty-two of the observed differences corresponded to proteins localized to the corpus in uninfected animals that were increased or decreased in abundance in WT-infected animals with the premalignant condition atrophic gastritis, as reported in a previous study (49). The current study analyzed proteins localized throughout the stomach instead of focusing exclusively on corpus-specific proteins. Therefore, of the 1,312 differences detected in the current study, 1,130 were distinct from the previously reported alterations in corpus-specific proteins (Table S7) (49).

**Fig 5:**
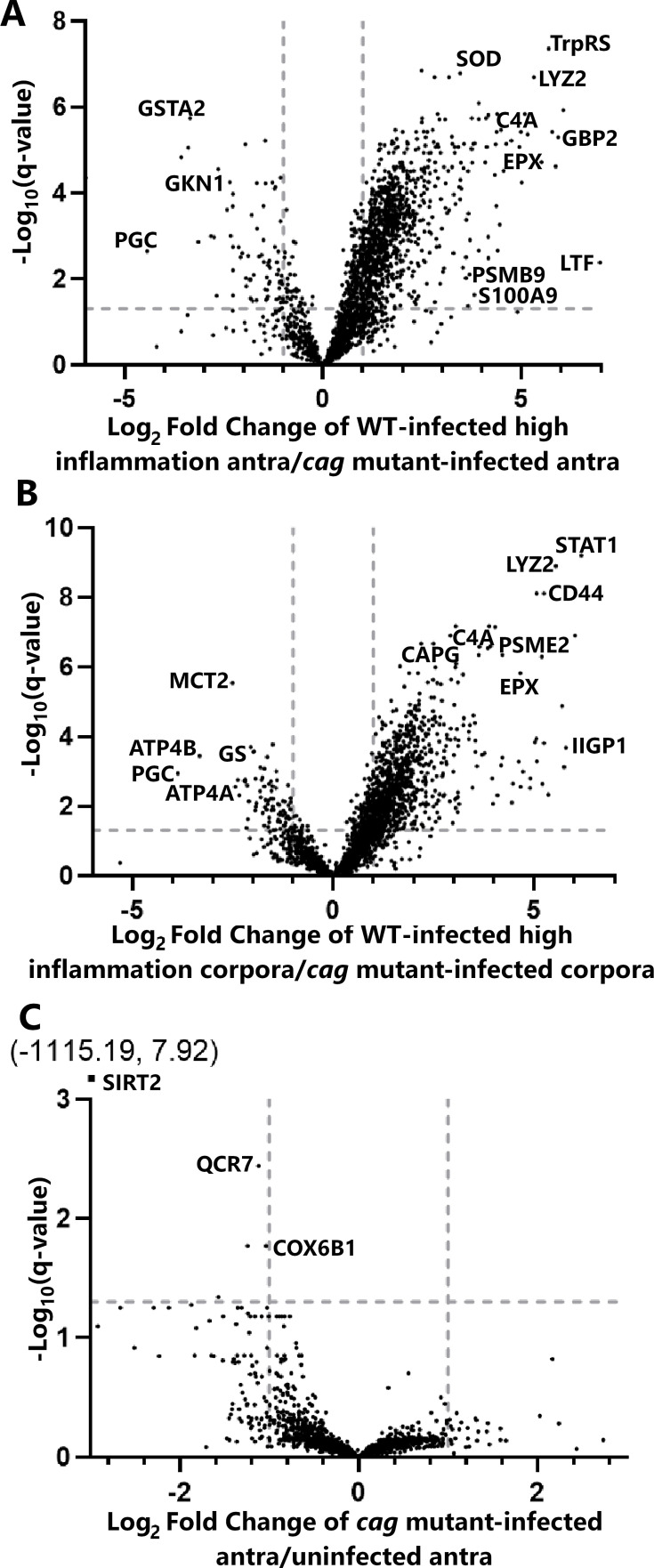
LC-MS/MS analysis reveals that *H. pylori-*induced gastric remodeling is driven by Cag T4SS-induced inflammation. (**A**) Hundreds of proteins are increased, and dozens are decreased in abundance in WT-infected antra with high inflammation compared to *cag* mutant-infected antra. (**B**) Hundreds of proteins are increased, and dozens are decreased in abundance in WT-infected corpora with high inflammation compared to *cag* mutant-infected corpora. (**C**) Five proteins are decreased in abundance in *cag* mutant-infected antra compared to uninfected antra. One protein (SIRT2) fell outside of the axis bounds and is denoted in text to the left of the volcano plot. The horizontal dashed line indicates a *P*-value of 0.05, and the vertical dashed lines indicate a fold change of −2 or 2. (**A and B**) Proteins with markedly decreased levels in WT-infected stomachs compared to *cag* mutant-infected stomachs include glutathione S-transferase alpha 2 (GSTA2), gastrokine 1 (GKN1), pepsinogen C (PGC), subunits of gastric H^+^/K^+^ ATPase (ATP4A and ATP4B), monocarboxylate transporter (MCT2), and glutamine synthetase (GS). Proteins with markedly increased levels include superoxide dismutase (SOD), tryptophanyl tRNA synthetase (tryptophan transfer RNA ligase, TrpRS), lysozyme 2 (LYZ2), complement component 4A (C4A), guanylate-binding protein 2 (GBP2), eosinophil protein X (EPX), proteasome components (PSMB9 and PSME2), lactotransferrin (lactoferrin, LTF), a calprotectin component (S100A9), capping actin protein (CAPG), interferon-inducible GTPase 1 (IIGP1), signal transducer and activator of transcription 1 (STAT1), and a cell-surface glycoprotein (CD44). Complete data are shown in Tables S5 to S11.

Considering the dependence of gastric transcriptional remodeling on the presence of inflammation, we next divided the WT-infected group into two subgroups: animals with high inflammation scores (>7) and those with low inflammation scores (<3). When comparing stomachs from WT-infected animals with low inflammation to stomachs from the *cag* mutant-infected animals, there were no proteomic differences detected in either the corpora or antra. When comparing the stomachs of WT-infected animals with high inflammation scores to corresponding regions from the *cag* mutant-infected animals, we detected an altered abundance of hundreds of proteins. Specifically, the abundance of 1,020 proteins was significantly increased, and the abundance of 74 proteins was significantly decreased in the antra of WT-infected animals with high inflammation scores (Fig. 5A; Table S8). The abundance of 939 proteins was increased, and the abundance of 85 proteins was decreased in the corpora of WT-infected animals with high inflammation scores compared to corpora from the *cag* mutant-infected animals (Fig. 5B; Table S9).

Among the differentially abundant proteins described above, several corresponding transcript sequences were analyzed with the previously described NanoString panel. We identified 13 transcripts and corresponding proteins that were significantly different in abundance in both the transcriptional analysis and the LC-MS/MS proteomic analysis (both antrum and corpus) when comparing tissues from WT-infected animals with high inflammation scores and *cag* mutant-infected animals (Table S10). For example, the parietal cell potassium voltage-gated channel subfamily E member 2 protein (KCNE2), involved in gastric acid secretion, was decreased in abundance at both the transcript and proteomic level when comparing WT-infected animals with high inflammation to *cag* mutant-infected animals (Tables S2, S9 and S10; Fig. 4A and 5B). Similarly, CD36 (platelet glycoprotein 4, a protein that contributes to gastric tissue repair) (55) and smoothelin (a marker for smooth muscle cells) were decreased in abundance in WT-infected animals with high inflammation. Proteins with significantly increased abundance at the transcriptional and proteomic levels when comparing WT-infected animals with high inflammation to *cag* mutant-infected animals included the calprotectin subunit S100A9 (a neutrophil component), two proteasome components (PSMB8 and PSMB9), guanylate-binding protein 2 (GBP2, an interferon-inducible GTPase) (51), CD44 (gastric cancer stem cell marker) (56, 57), HLA class II histocompatibility antigen gamma chain (CD74, a cell-surface receptor for *H. pylori* adhesion) (58), keratin type II cytoskeletal 7 (KRT7) (59, 60), polymeric immunoglobulin receptor (PIGR, which transports polymeric Ig [IgA or IgM] from the basolateral surface of the epithelium to the apical side), immunity-related GTPase family M protein 1-like (IRGM, which regulates autophagy), and peptidyl-prolyl cis-trans isomerase FKBP5 (which modulates glucocorticoid signaling; Tables S2, S8 to S10; Fig. 4A, 5A and B). The transcriptional and proteomic alterations in S100-A9 correspond to the increased presence of neutrophils detected by histologic analysis (acute inflammation; Fig. 2), while reduced KCNE2 corresponds to the decreased presence of visible parietal cells in stomachs from WT-infected animals with high inflammation.

Many of the proteins exhibiting markedly increased abundance in WT-infected animals with high inflammation scores have roles in the innate and adaptive immune response. Markers of an innate immune response included the antimicrobial iron-binding protein lactotransferrin (lactoferrin), myeloperoxidase, lysozyme C2, and eosinophil peroxidase, which were all increased fivefold or more in WT-infected animals with high inflammation compared to *cag* mutant-infected animals (Fig. 5A and B; Tables S8 and S9). As expected, markers of specific immune cells, including neutrophil cytosol factor 1, T-cell specific GTP binding protein 1-like protein, and B-cell marker CD20, were also increased in WT-infected animals with high inflammation (Table S8 and S9).

Proteins that were decreased in abundance in WT-infected animals with high inflammation scores compared to *cag* mutant-infected animals included gastrokine 1 (GKN), which is produced by gastric epithelial cells and has key roles in maintenance of cell proliferation, differentiation, and mucosal homeostasis (61–63); ATP4A and ATP4B, essential subunits of the parietal cell-specific ATPase; and PGC, the precursor protein to the digestive gastric enzyme gastricsin (Fig. 5A and B; Tables S8 and S9).

To identify *H. pylori-*induced gastric alterations independent of Cag T4SS or CagA functionality, we compared the proteomic results from *cag* mutant-infected animals to corresponding results from uninfected animals. Surprisingly, there were no differences detected when comparing the corpora, and only five differences detected when comparing the antra, all of which were decreased in *cag* mutant-infected antra compared to uninfected antra (Fig. 5C; Table S11).

### Imaging mass spectrometry of gastric tissues reveals gastric proteomic alterations dependent on Cag T4SS activity

**Fig 6:**
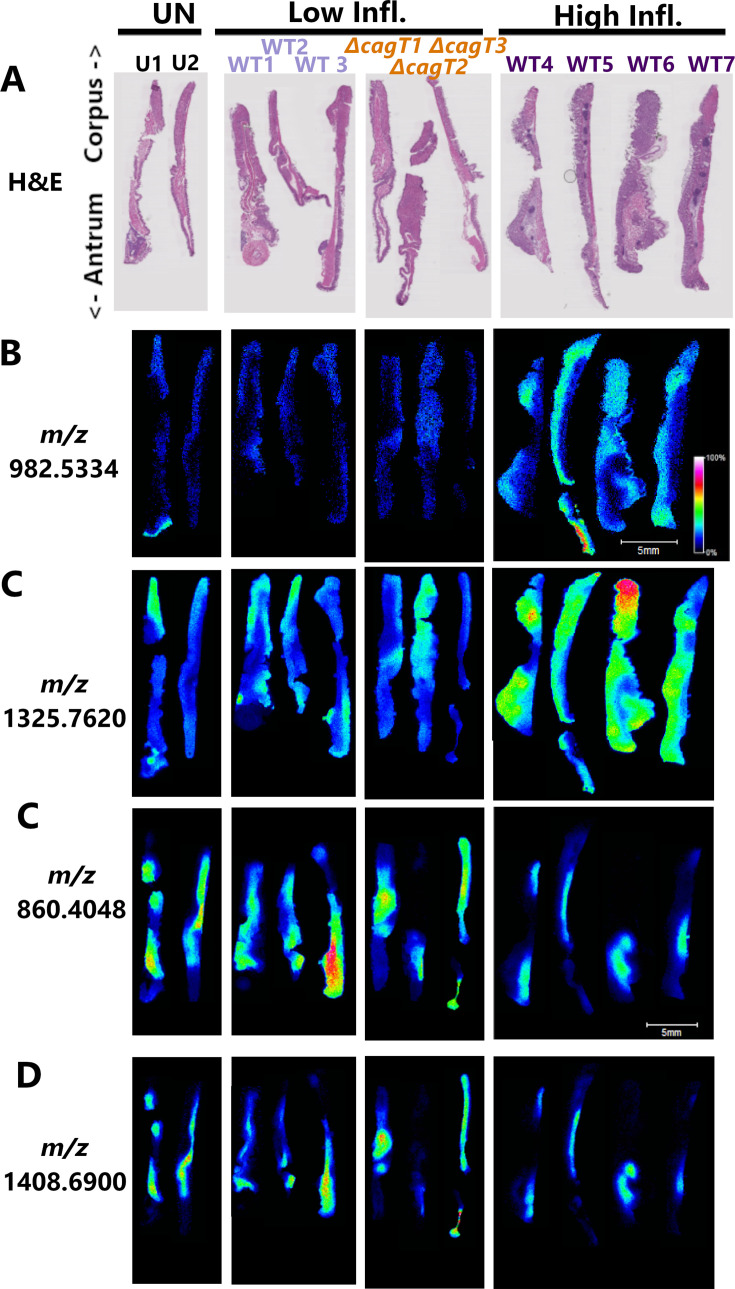
IMS analysis depicting tryptic peptides with increased or decreased abundance in animals with high inflammation compared to uninfected animals or infected animals with low inflammation. Tissues are oriented with the corpus at the top and the antrum at the bottom. Infection/inflammation state labels are shown above the images (UN, uninfected; Low Infl., low inflammation; High Infl., high inflammation). Individual tissues are labeled as follows: U, uninfected; WT, infected with wild-type *H. pylori*; *ΔcagT*, infected with the *ΔcagT* mutant strain. The figure shows *m/z* values for each tryptic peptide. Putative identifications are provided in Table S12. (**A**) H&E stains of gastric tissue from uninfected and infected animals. (B–E) Ion images showing localization of peptides *m/z* 982.5334, matched to vasodilator-stimulated phosphoprotein isoform X1 and/or sodium/potassium-transporting ATPase subunit alpha-1(B); *m/z* 1325.7620, matched to cullin-associated NEDD8-dissociated protein 1 and/or inosine-5’-monophosphate dehydrogenase 2 (**C**); *m/z* 860.4048, matched to huntingtin-interacting protein 1-related protein (**D**); and *m/z* 1408.6900, matched to transgelin (**E**). Scale bar: 5 mm. The figure is a compilation of multiple tissue images joined together in a different order than that of the original slides.

**Fig 7:**
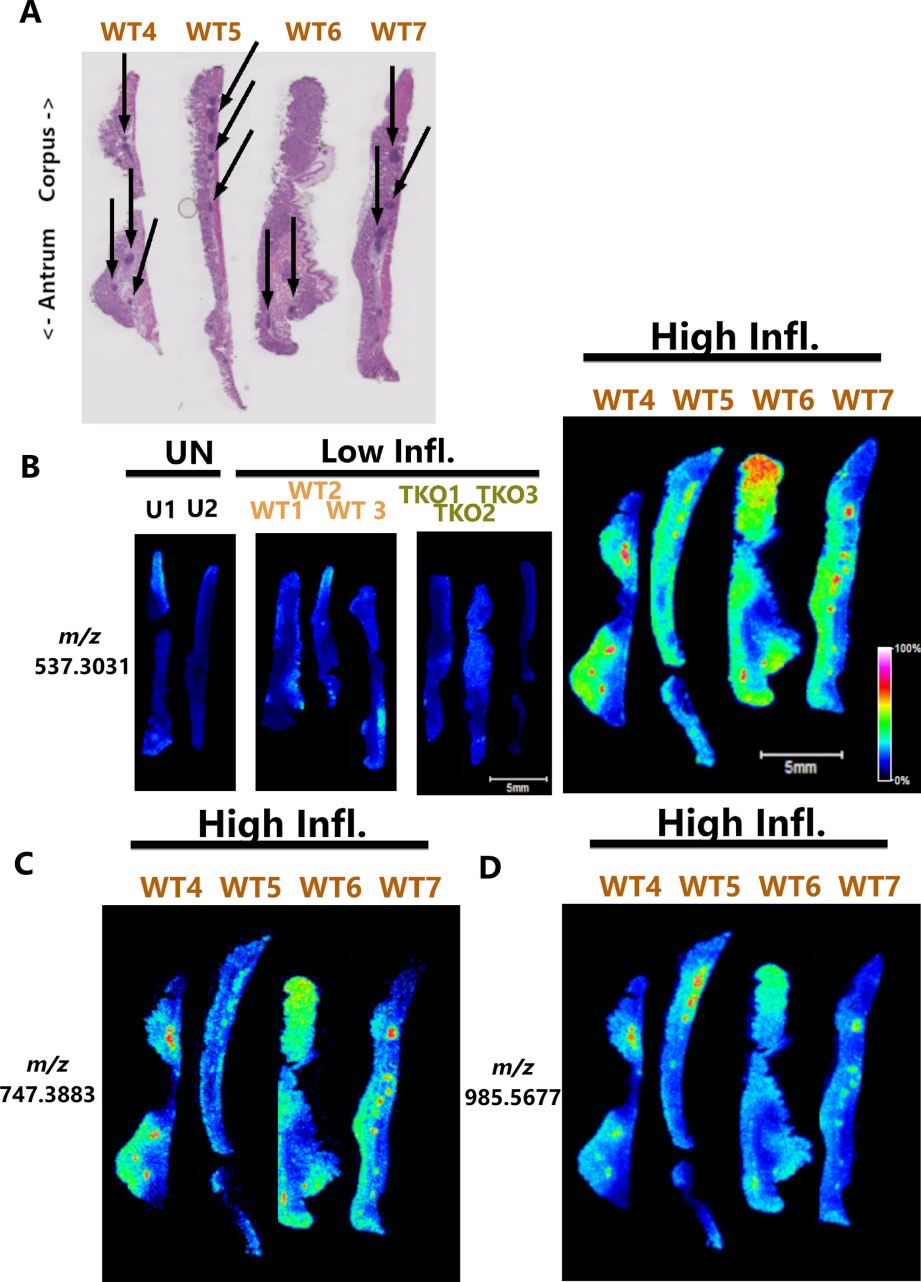
IMS analysis depicting tryptic peptides with increased abundance in lymphoid follicles. Tissues are oriented with the corpus at the top and the antrum at the bottom. Infection/inflammation state labels are shown above the images (UN, uninfected; Low Infl., low inflammation; High Infl., high inflammation). Individual tissues are labeled as follows: U, uninfected; WT, infected with wild-type *H. pylori; ΔcagT*, infected with the *ΔcagT* mutant strain. (**A**) H&E stains of gastric tissue from WT-infected animals with high inflammation, with black arrows indicating lymphoid follicles. The H&E-stained images in **Fig. 7A** are identical to the corresponding images in Fig. 6A. (**B**) Ion images showing localization of peptide *m/z* 537.3031 in the gastric tissues. (**C and D**) Enlarged ion images showing localization of peptides *m/z* 747.3883 (**C**) and *m/z* 985.5677 (**D**) in the WT-infected tissues that developed high inflammation. Scale bar: 5 mm. The figure is a compilation of multiple tissue images joined together in a different order than that of the original slides.

In conjunction with the LC-MS/MS experiments described above, we used matrix-assisted laser desorption/ionization imaging mass spectrometry (MALDI IMS) to analyze the gastric tissues. This approach has a lower sensitivity than LC-MS/MS but allows the localization of proteins within tissues to be determined. Because MALDI IMS has a higher sensitivity for detecting peptides instead of intact proteins, we sprayed the tissues with trypsin and then imaged the resulting peptides. For these experiments, we analyzed stomach sections (including both corpus and antrum regions) from the WT-infected animals (with high or low inflammation), *ΔcagT* mutant-infected animals, and uninfected animals. Images were analyzed to detect tryptic peptides that differed in abundance or localization when comparing infected animals to uninfected animals, *ΔcagT* mutant-infected animals to other groups, or infected animals with high inflammation to other groups. Proteins that were corpus specific in uninfected animals and altered in WT-infected animals with atrophic gastritis were reported in a previous study (49) and therefore not reported in the current study. Instead, we focused on identifying proteins exhibiting either increased or decreased abundance throughout the stomach or focal increases in signal intensity. This analysis identified 24 peptides that were differentially abundant in highly inflamed gastric tissues of WT-infected animals compared to tissues with minimal or no inflammation (Fig. 6 and 7; Table S12). Twelve peptides were diffusely increased in abundance throughout the stomach in WT-infected animals with high inflammation compared to WT-infected animals with low inflammation, Δ*cagT* mutant-infected animals (all of which exhibited low inflammation), and uninfected animals. Representative images of peptides with this pattern are shown in Fig. 6. Four peptides exhibited increased abundance in specific foci in infected animals with high inflammation compared to uninfected animals or infected animals with low inflammation. Representative images of these peptides are shown in Fig. 7. Interestingly, comparison of these punctate spots of signal intensity with H&E-stained images of serial tissue sections revealed that the areas of high signal intensity co-localized with lymphoid follicles in the highly inflamed tissues (indicated by black arrows in Fig. 7A). Finally, the abundance of eight peptides was diffusely decreased throughout the stomach in infected animals with high inflammation compared to infected animals with low inflammation and uninfected animals (Fig. 6). In some cases, infected animals with low inflammation exhibited a peptide abundance that was intermediate between that of animals with high inflammation and uninfected animals, and in others, the distribution was more comparable to uninfected animals. In all cases, the results for Δ*cagT* mutant-infected animals were virtually indistinguishable from the results for the WT-infected animals with low inflammation.

We cross-referenced the IMS data with LC-MS/MS data to determine the putative molecular identities of the 24 differentially abundant peptides (Table S12). For example, tryptic peptides from huntingtin-interacting protein 1-related protein (*m/z* 860.4048, involved in vesicular trafficking) and transgelin (*m/z* 1408.69, an actin-binding protein) were decreased in abundance in stomachs from WT-infected animals with high inflammation compared to stomachs from other groups of animals (Fig. 6). In cases where peptides matched to multiple potential protein identifications (indicated by adjacent rows with the same color highlighting and near-identical *m/z* values in Table S12), both putative matches might account for the IMS pattern with the observed *m/z* value, or alternatively, only one may be relevant. Because trypsin solution was applied by hand to entire corporal or antral regions of the stomach (instead of being specifically applied to lymphoid follicles), it was not possible to confidently identify the four peptides that were increased specifically in lymphoid follicles (Fig. 7) using the current experimental approach.

### Pathway analysis highlights Cag T4SS-dependent alterations in the gastric proteome

**Fig 8:**
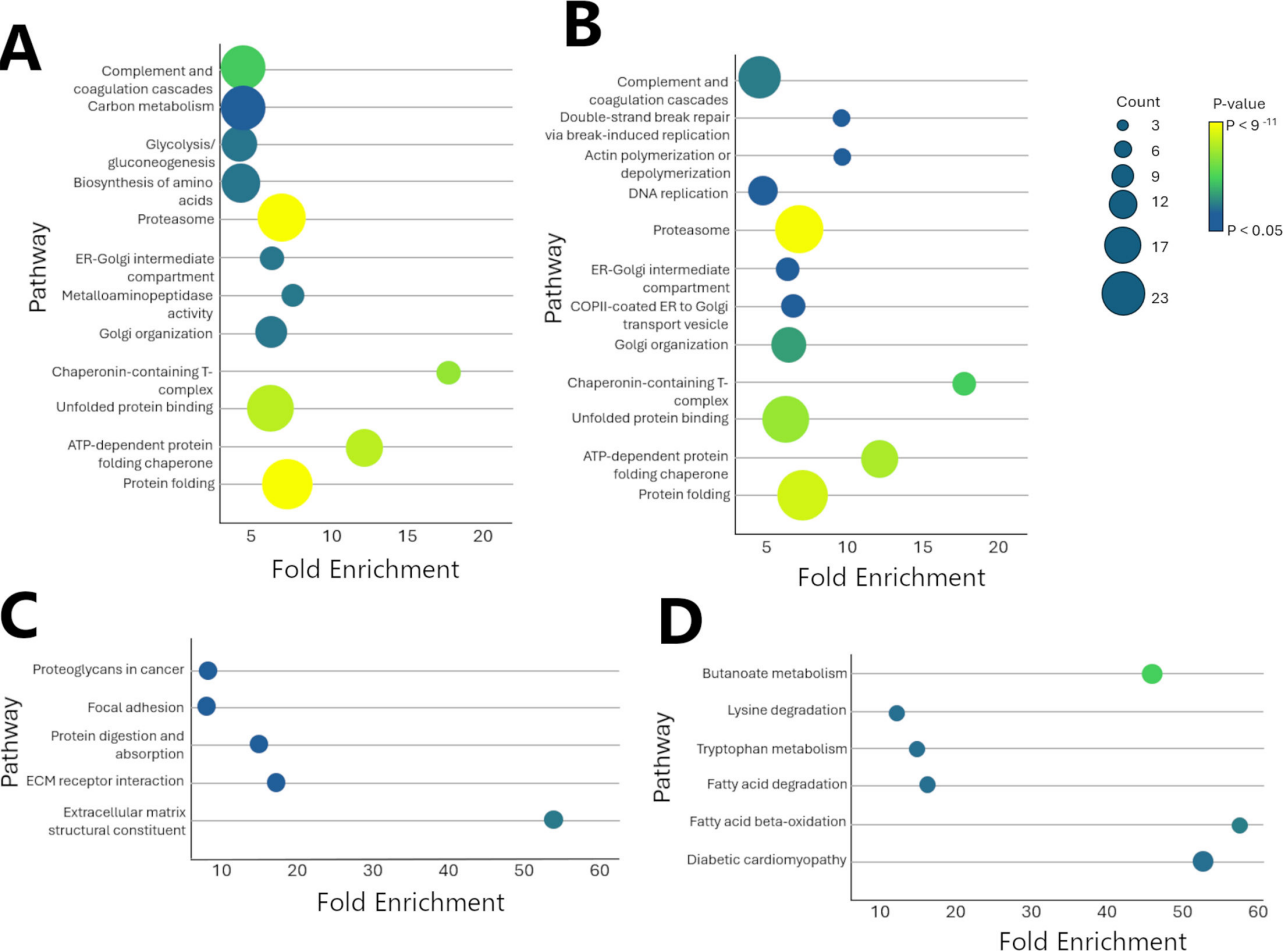
Pathways associated with Cag T4SS-dependent remodeling of the gastric proteome. Proteins that were differentially abundant in WT-infected animals with high inflammation compared to *cag* mutant-infected animals were analyzed to identify relevant enriched pathways. (A) Enriched pathways corresponding to proteins that are increased in abundance in the antrum. (**B**) Enriched pathways corresponding to proteins that are increased in the corpus. (**C**) Enriched pathways corresponding to proteins that are decreased in the antrum. (**D**) Enriched pathways corresponding to proteins that are decreased in the corpus. The terms “count” and “fold enrichment” are defined in Materials and Methods. Related data are shown in Table S13.

To better understand the functional effects of observed alterations in the gastric proteome, we performed pathway analyses of the LC-MS/MS data. In brief, lists of proteins that were increased or decreased in abundance in the corpus or antrum when comparing WT-infected animals with high inflammation to *cag* mutant-infected animals were analyzed using the Database for Annotation, Visualization, and Integrated Discovery (DAVID) bioinformatic resource system. Examples of pathways demonstrating statistically significant enrichment are shown in Fig. 8. For example, independent proteomic analyses of the antrum and corpus showed that numerous differentially abundant proteins (increased abundance in WT-infected animals with high inflammation) mapped to complement/coagulation cascades, the proteasome pathway, and pathways related to protein folding. Nine complement components or complement-regulatory components were differentially abundant (Table S13). Differentially abundant proteins mapping to the coagulation cascade included fibrinogen components (FGA, FGB, and FGG), SERPINC1 (antithrombin), and F2 (prothrombin). Numerous proteasome components (*n* = 21) were differentially abundant, including proteasome subunits beta types 8 and 9 (PSMB8 and PSMB9), a result corroborated by NanoString transcriptional analysis (Fig. 4).

## DISCUSSION

Some clinical isolates of *H. pylori* contain a pathogenicity island (*cag* PAI) that encodes components of a Cag T4SS and the secreted effector protein CagA, while other strains do not (16, 17). Persistent colonization of the human stomach with *cag* PAI-positive *H. pylori* strains has been associated with an increased risk of developing gastric cancer (compared to colonization with *cag* PAI-negative strains) (17). In this study, we infected Mongolian gerbils with a WT *cag* PAI-positive strain or mutant strains in which functionally important *cag* PAI genes were deleted (*ΔcagA*, *ΔcagT*, or *ΔcagY*) and analyzed the gastric histologic, transcriptional, and proteomic alterations that occurred in response to each of the strains.

The WT strain and each of the *cag* mutant strains of *H. pylori* were able to colonize Mongolian gerbils, with colonization rates ranging from 80% to 95% (Fig. 1). Only the WT strain of *H. pylori* was able to cause high levels of inflammation and severe gastric disease. The presence of a robust gastric inflammatory response was associated with extensive transcriptional and proteomic remodeling of the stomach. In contrast, the *cag* mutants induced minimal gastric inflammation (i.e., low inflammation scores or no detectable inflammation; Fig. 1 and 2), and there were minimal gastric transcriptional or proteomic alterations in the mutant-infected animals. Lack of complementation is a limitation of this study, but the testing of two Cag T4SS-negative mutants (*ΔcagT* and *ΔcagY*) with similar phenotypic properties mitigated this limitation.

Considering the different properties of the *ΔcagA* mutant compared to the *ΔcagT* and *ΔcagY* mutants *in vitro* (Fig. S1), we hypothesized that there might be detectable differences in the capacity of these mutants to alter gastric transcriptional or proteomic profiles *in vivo*. In other words, we anticipated that it would be possible to differentiate *cag* PAI-dependent effects requiring CagA from *cag* PAI-dependent, CagA-independent effects linked to the T4SS-mediated delivery of non-protein pathogen-associated molecular patterns (such as LPS metabolites or peptidoglycan) into gastric cells *in vivo*. Notably, we did not detect any significant differences in the gastric transcriptional or proteomic profiles of animals infected with the CagA mutant compared to those from animals infected with Cag T4SS mutants. This implies that there are differences in responses to *H. pylori* in the AGS co-culture assay compared to gastric responses in the Mongolian gerbil model. Specifically, NF-kB was activated by CagA-negative/Cag T4SS-positive mutants but not Cag T4SS-negative mutants *in vitro* (Fig. S1A), but there were no significant differences in the abundance of transcripts or proteins when comparing the gastric transcriptional or proteomic profiles of Mongolian gerbils infected with the CagA-negative/Cag T4SS-positive mutants to the Cag T4SS-negative mutants.

WT-infected animals developed gastric inflammation in a biphasic pattern, with about 33% developing minimal or no inflammation and about 67% developing high levels of inflammation and gastric disease (Fig. 1; Fig. S2). This variation in the gerbil model mimics the variation in gastric outcomes observed in humans infected with *H. pylori*. We speculate that the variation observed in the current study may reflect differences in how the outbred Mongolian gerbils’ immune systems respond to *H. pylori*. Other possible explanations include *H. pylori* mutations leading to the loss of Cag T4SS activity in some animals and/or differences among animals in the numbers or location of *H. pylori* throughout the stomach. The WT-infected animals exhibiting low levels of gastric inflammation had gastric transcriptional and proteomic profiles similar to those of *cag* mutant-infected animals and uninfected animals. This highlights the capacity of *H. pylori* to colonize the stomach without eliciting a substantial host response.

The rate of recovery for culturable *H. pylori* was lower for WT-infected animals than for mutant-infected animals (Fig. 1B). Among the 14 WT-infected animals from which bacteria were not successfully cultured at the endpoint of infection, 11 exhibited inflammation scores >7 (Fig. S2). Conversely, of the 20 WT-infected animals with high gastric inflammation and disease, 11 did not have culturable bacteria at the endpoint of infection. Steiner staining allowed the detection of *H. pylori* in many of the infected animals with negative culture results. The lack of culturable bacteria from stomachs with high inflammation could be due to multiple factors, including reduced numbers of *H. pylori* in highly inflamed stomachs or the presence of gastric antibacterial molecules that inhibit bacterial growth when stomach homogenates are cultured. If high inflammation scores are considered evidence that *H. pylori* initially colonized the stomach, then only 3 out of 30 WT-infected animals were not detectably infected with *H. pylori*. This indirect evidence of *H. pylori* colonization yielded the same colonization rate for the WT strain (the only strain that caused high inflammation scores) as CFU data coupled with the Steiner staining approach.

In previous studies, we reported on corpus-specific molecular alterations induced by a WT *H. pylori* strain in Mongolian gerbil gastric tissues exhibiting the premalignant condition atrophic gastritis (47, 49). These included loss of proteins or lipids normally localized to the corpus in healthy, uninfected stomachs. In the current study, we analyzed proteomic alterations throughout the stomach (not limited to the corpus), which allowed us to detect numerous alterations not reported in the previous study. When comparing the gastric transcriptional profiles of WT-infected animals that developed high inflammation scores to profiles of other groups, alterations were consistent with an increased Th1/Th17 response (for example, increased levels of *Il1b*, *Tbx21*, *Il21*, and *Tgfb1*). LC-MS/MS analysis of the gastric tissues revealed a differential abundance of more than 1,000 proteins when comparing stomachs from WT-infected animals with high inflammation scores and stomachs from *cag* mutant-infected animals. Proteins with increased abundance were associated with both innate (myeloperoxidase, lysozyme C-2, and immunoproteasome subunits) and adaptive immune responses (HLA class II histocompatibility antigens and lymphocyte-specific proteins). We also detected a decreased abundance of proteins with roles in the cell cycle (various histone proteins and other DNA-binding proteins), cell proliferation (proliferation-associated protein 2G4, proliferating cell nuclear antigen), and maintenance of gut mucosal homeostasis (components of the parietal cell-specific ATPase and various gastric hormones and enzymatic precursors).

Independent analysis of proteomic results from gastric corpora and antra revealed several enriched pathways in common, including the complement/coagulation cascade and the proteasome pathway (Fig. 8A and B; Table S13). The observed alterations in the complement/coagulation cascade are consistent with an activated gastric mucosal inflammatory response, which may reflect both a host response to *H. pylori* and a host response to gastric mucosal injury (64). Alterations in proteasome pathway function are relevant to gastric cancer, as proteasome systems have been shown to modulate gastric cancer tumor microenvironments, and proteasome typing can be used to stratify gastric cancer risk (65–67). Increased proteasome levels can also be a marker of antigen-presenting cells recruited to the tissue. Additionally, proteasome inhibitors are currently of interest for cancer therapy (65). Interestingly, there was no overlap in the enriched pathways for proteins that were decreased in abundance in the corpus or antrum. This suggests that there are both shared and unique facets of the gastric proteomic response to the presence of an active Cag T4SS in different parts of the stomach.

From a mechanistic perspective, the CagA- and Cag T4SS-dependent gastric alterations detected in this study likely resulted from multiple processes. An increased abundance of some proteins or transcripts is attributable to the influx of immune cells into inflamed gastric tissues. For example, increased abundance of calprotectin components is attributable to an influx of neutrophils. Other CagA-dependent gastric alterations can be attributed to either indirect effects of gastric inflammation on gastric tissues or direct effects of *H. pylori* on gastric mucosal tissue. Many of the *cag* PAI-dependent gastric alterations detected in this study can be linked to the presence of gastric hyperplasia (characterized by thickening of the gastric mucosal layer), which is likely a consequence of both inflammation and direct stimulation of gastric stem cells by *H. pylori* (68–70). Finally, a CagA-dependent reduction in the abundance of some proteins or transcripts can be attributed to a loss of specialized cell types (parietal cells and chief cells) in the gastric corpus in stomachs exhibiting atrophic gastritis (47, 49) and/or reduced production of these proteins or transcripts.

Interestingly, IMS revealed co-localization of four peptides with some, but not all, lymphoid follicles (Fig. 7). This pattern suggests that there may be multiple different types of lymphoid aggregates in the highly inflamed gastric environment, or alternatively, the sections analyzed are from different areas of lymphoid aggregates (e.g., central or peripheral portions). The different protein signatures of these aggregates may indicate the presence of tertiary lymphoid structures (i.e., some may contain germinal centers) (71–73). Tertiary lymphoid structures have been studied in the context of inflammatory diseases and cancer and are associated with positive outcomes in diffuse-type gastric cancers (74–77). As *H. pylori* infection is associated with both intestinal and diffuse-type gastric cancers, the properties of these lymphoid structures (including the potential presence of characteristics of tertiary lymphoid structures) warrant further study.

Several of the differential abundant transcripts or proteins identified in this study have previously been reported to be relevant to cancer pathogenesis, markers for gastric premalignant lesions (spasmolytic polypeptide-expressing metaplasia, intestinal metaplasia, etc.), markers for gastric cancer, or markers for gastric cancer prognosis. For example, GBP2 (an interferon-inducible GTPase associated with neoplastic tissue and poor prognosis in gastric cancer) was increased in abundance in WT *H. pylori*-infected stomachs with high inflammation compared to stomachs from *cag* mutant-infected animals (51). GBP5, a protein detected by IMS that was more abundant in highly inflamed tissues than tissues with minimal or no inflammation (Table S12), is an activator of inflammasome assembly and has been implicated as a marker for multiple types of immunologically hot tumors or used to predict therapeutic outcomes for lung cancer patients (78, 79). Other proteins reported to be associated with carcinogenesis, such as CD44 (which is overexpressed in cancer stem cells [56]) and KRT7 (which is overexpressed in multiple cancer types [59, 60]) were also increased when comparing WT-infected animals with high inflammation to *cag* mutant-infected animals. Gastrokine 1 was decreased in abundance in WT-infected animals with high inflammation; this protein is considered a tumor suppressor, and reduced levels have been detected in patients with gastric cancer compared to patients without gastric cancer (62, 63). We speculate that many of the other differentially abundant transcripts or proteins detected in the current study may also have relevant roles in gastric cancer pathogenesis or might be markers for gastric carcinogenesis.

In summary, this study confirms that CagA and Cag T4SS activity are important determinants of gastric pathology, and we delineate transcriptional and proteomic alterations that are dependent on the actions of the Cag T4SS and CagA. In addition, we report that many *H. pylori*-infected gerbils (including a significant proportion of animals infected with the WT strain) had low inflammatory scores and no detectable transcriptional or proteomic alterations. This highlights the principle that clinically relevant *H. pylori* strains can potentially chronically colonize the stomach without inducing substantial host responses.

## MATERIALS AND METHODS

### *H. pylori* culture methods

*H. pylori* strain 7.13 is a derivative of the human clinical isolate B128 (80) that has been adapted to the Mongolian gerbil model. Rodent-adapted 7.13 has an intact *cag* pathogenicity island that encodes components of a functional Cag T4SS and the CagA oncoprotein, and it can cause gastric cancer in the Mongolian gerbil model (46, 49). Wild-type 7.13 and mutant strains (described below) were grown at 37°C on trypticase soy agar (TSA) plates with 5% sheep blood in room air containing 5% supplemental CO_2_. Prior to animal infection, strains were grown in bisulfite-free Brucella broth containing 10% fetal bovine serum (FBS) overnight in a shaking incubator under the same conditions.

### Generation and analysis of *H. pylori* mutant strains

The Mongolian gerbil-adapted wild-type strain 7.13 was used for mutagenesis. A ∆*cagT* mutant (designated 7.13_5.2∆*cagT*) was described previously (49). To generate ∆*cagY* and ∆*cagA* mutant strains (named 7.13_5.2*∆cag*Y and 7.13_5.2*∆cag*A, respectively), we designed a plasmid containing a chloramphenicol acetyltransferase gene flanked by 500 base pairs of *H. pylori* genomic sequences on either side of the gene to be deleted. After the plasmid was transformed into strain 7.13, transformants were selected by growth on sodium bisulfite-free Brucella agar plates supplemented with 2.5% fetal bovine serum and 5 µg/mL chloramphenicol.

PCR analysis confirmed the deletion of the appropriate gene in the transformants. To confirm deletion of the target protein, Western blots for CagA, CagT, and CagY were performed, using rabbit polyclonal antisera (Fig. S1) (81). To evaluate Cag T4SS activity, an NF-κB assay was performed (82) (Fig. S1). In short, AGS NF-kB luciferase reporter cells (82) were incubated with the indicated strains for 2.5 hours, and then relative luminescence was measured. The wild-type parental strain exhibited intact Cag T4SS activity (Fig. S1). The *∆cagA* mutant strain retained Cag T4SS activity, whereas the *∆cagT* and *∆cagY* mutant strains were inactive in this assay (Fig. S1).

### Mongolian gerbil experiments

Male and female Mongolian gerbils (35–50 g weight) were obtained from Charles River Laboratories. About a week after arriving in the facility, the animals’ diet was changed to AIN-93M chow (Bio-Serv), which is associated with an increased incidence of gastric disease (42, 47). One day prior to infection, Mongolian gerbils were fasted overnight. The next day, animals were orally gavaged with a 500 µL suspension (1 × 10^9^ CFU/mL in Brucella broth without FBS) of WT *H. pylori* strain 7.13, a pool of three to five Δ*cagA*, Δ*cagT*, or Δ*cagY* mutant single colonies, or Brucella broth. The oral gavage was repeated on day 2. After 12 weeks, the animals were euthanized and dissected as described below.

We transcriptionally and proteomically analyzed eight stomachs infected with the WT strain (four with high inflammation and four with low inflammation) and 4–5 stomachs from each of the other groups. All the stomachs from *H. pylori*-infected animals selected for analysis were culture-positive and/or exhibited high gastric inflammation scores (Fig. 1 and 2). *H. pylori* and gastric inflammation were not detected in any of the uninfected animals, so uninfected stomachs were selected for analysis at random.

Tissues from a subset of the Mongolian gerbils analyzed in the current study have been analyzed previously; specifically, 2–4 of the WT-infected, Δ*cagT* mutant-infected, and uninfected animals were used in a previous study to analyze proteins that are corpus specific in uninfected animals, using LC-MS/MS and IMS (49). The previous study focused on proteins that were restricted to the corpus in uninfected Mongolian gerbil stomachs and decreased or delocalized in the premalignant condition atrophic gastritis and did not include analysis of animals infected with CagA or CagY mutants. The LC-MS/MS proteomic analysis results reported in the current study are based on a new independent analysis (distinct from the analysis reported in a previous manuscript), which analyzed gastric tissues from all groups (WT-infected, mutant-infected, and uninfected) at the same time. Though the current study uses some of the same animal tissues that were previously analyzed, the current study focuses on different biological findings, reporting peptides that are diffusely increased or decreased in the stomach or increased specifically in lymphoid follicles. The transcriptional analysis reported in this study used tissues from an animal cohort that has not previously been transcriptionally profiled.

### Processing of gastric tissue

Mongolian gerbil stomachs were dissected to remove the nonglandular forestomach. The glandular region (including the corpus and antrum) was cut along the lesser curvature and laid flat for processing. The flattened stomach was cut into four longitudinal strips, such that each strip contained corporal and antral tissue. Surgical dye was used to mark the corpus of the largest strip (corresponding to about one-third to half of the glandular stomach), which was then placed in a plastic cassette wrapped with aluminum foil, flash-frozen in dry ice, and stored at −70°C; this strip was subsequently used for IMS and LC-MS/MS analysis. Another portion of the glandular stomach was fixed in 10% neutral-buffered formalin, embedded in paraffin, and the histology of the formalin-fixed paraffin-embedded tissues was analyzed as described below. The third strip was flash frozen in liquid nitrogen for subsequent RNA analysis, and the last strip was utilized for bacterial culture, both of which are described below.

### Bacterial density analysis

One longitudinal strip of stomach was homogenized in 1 mL of Brucella broth with 10% FBS for 1 min using a Bead Ruptor 12 Homogenizer Bullet Blender (Omni). One hundred microliter aliquots of undiluted homogenate were cultured on TSA plates supplemented with 5% sheep blood, nalidixic acid (10 µg/mL), vancomycin (50 µg/mL), amphotericin (20 µg/mL), and bacitracin (100 µg/mL). Ten microliter aliquots of logarithmic dilutions (10^−1^, 10^−2^, 10^−3^, and 10^−4^) were plated on one such TSA plate and tilted to allow for a streak of bacterial growth (83); these dilution plates were used to enumerate bacterial density.

### Gastric histologic analysis

Strips of the stomach containing both corpus and antrum were fixed in 10% neutral-buffered formalin for about 24 hours. Each tissue was then embedded in paraffin, sectioned onto a slide, and stained with H&E. A gastrointestinal pathologist examined the H&E-stained sections of gastric tissues in a blinded fashion. Scores (0, 1, 2, and 3, corresponding to absent, mild, moderate, or marked inflammation, respectively) were assigned to evaluate both acute (neutrophils/polymorphonuclear leukocytes) and chronic (mononuclear leukocytes) inflammation in the antrum and corpus, resulting in a total inflammation score of 0–12 (84). Numbers of lymphoid aggregates were counted along a single longitudinal strip of tissue, including the corpus and antropyloric region. For animal stomachs from which *H. pylori* was not cultured at the endpoint of infection, Steiner stains were performed to detect *H. pylori* within the gastric tissue.

### Analysis of gastric transcriptional profiles using a custom Mongolian gerbil NanoString panel

RNA was extracted from whole strips of stomach tissue, including both the corpus and antrum, using the acid-guanidinium-phenol (Trizol)-chloroform method as previously described (49). Extracted RNA was analyzed in a quantitative multiplex nucleic acid hybridization assay using a previously described custom-designed Mongolian gerbil NanoString panel (NanoString Technologies, Seattle, WA) (49). This custom NanoString panel included 148 genes of interest across 27 functional categories. As the NanoString panel was primarily designed to evaluate the gastric mucosal inflammatory response, the genes included on this panel are not representative of the entire genome. Resources from the Vanderbilt Technologies for Advanced Genomics core were used to hybridize and process samples on the nCounter prep station, and the NanoString digital analyzer was used to generate Reporter Code Count raw data files.

Data files were normalized and processed as previously described (49). For comparisons visualized with volcano plots, a Benjamini-Hochberg multiple test correction was used to generate a corrected *P*-value (*q*-value). The volcano plot data were visualized using GraphPad. The transcriptional profiling reported here is from an entirely different animal cohort than has previously been transcriptionally analyzed and reported (49).

### Slide preparation and processing for mass spectrometry analysis

Fresh frozen Mongolian gerbil stomach tissues were prepared and washed to remove salts and lipids as previously detailed (49, 85). Not all tissue samples could fit on a single slide, so they were distributed onto two slides. Each slide contained uninfected tissues and infected tissues from each group. Uninfected and infected tissues were always sectioned, prepared, and analyzed together. Imaging mass spectrometry and LC-MS/MS analyses were done on serial sections of the same tissues.

### MALDI IMS analysis of peptides

Tissues were washed and Trypsin-digested overnight as previously described (85). For matrix application, 5 mg/mL α-cyano-4-hydroxycinnamic acid in 90% acetonitrile, 0.1% TFA was applied using an HTX M3 TM sprayer set to eight passes, 85°C nozzle, 700 mm/min, 45°C stage, and 2 mm track spacing. Peptide images were obtained on a 15 Tesla Bruker SolariX FT-ICR mass spectrometer in positive ion mode. Tryptic peptide images from *m/z* 500 to 3,500 were acquired using a time-domain file size of 512 K, resulting in an ICR transient length of 0.5767 s and a resolving power of ~65,000 at *m/z* 1,200 with 75-micron lateral resolution, 100 shots per pixel, medium laser focus, and no random walk.

### Analysis of MALDI data

We used flexImaging software (Bruker Daltonics) to manually analyze each peak in the mass spectrum and thereby identify signals of interest; no automated algorithms or supplemental analysis programs were used. Total ion current normalization was used to normalize imaging patterns. The grouping of tissues shown in figures is not identical to the organization of the original slides, which were laid out with the tissues mixed together (not grouped by infection or disease status) to minimize bias.

### LC-MS/MS sample preparation

Peptides were micro-extracted as previously described (49). In brief, following overnight trypsin digestion (85), peptides were micro-extracted from the corpus or antrum, dried in a Speedvac with no heat, then stored at −80°C until ready for LC-MS/MS analysis. Samples were reconstituted in 10 µL 0.2% formic acid, and 1 µL was injected using a 0.3 × 0.5 mm C18 trap column (ThermoFisher) and resolved using a 1-hour aqueous to the organic gradient on a 150 µm × 25 cm reversed-phase column (PepSep - Bruker). Peptides were resolved using a NanoElute2 (Bruker) and ionized via a captive spray source into a timsTOF HT (Bruker). Data were acquired via data-dependent PASEF using trapped ion mobility separation with 10 MS/MS ramps per MS1 cycle with a target intensity of 20,000 and an intensity threshold of 2,500.

### LC-MS/MS data analysis

LC-MS/MS results were analyzed using FragPipe (software for FragPipe, a complete proteomics pipeline with the MSFragger search engine at heart, can be found at nesvilab.org). For identifications and MS1 quantitation via IonQuant (86), results were searched against a Mongolian gerbil protein database downloaded from NCBI-RefSeq appended to an *H. pylori* protein database, with default parameters for LFQ-MBR searches performed using MSFragger (87). Statistical comparisons among groups (uninfected, WT-infected with high inflammation, WT-infected with low inflammation, *ΔcagA* mutant strain-infected, *ΔcagT* mutant strain-infected, and *ΔcagY* mutant strain-infected) and between gastric regions (antrum and corpus) were performed using MSStats (88). Search results filtered to a 1% false discovery rate using Percolator (89) were also loaded into Scaffold (Scaffold | Proteome Software, Inc., Portland, OR) for visualization and comparisons.

### Matching IMS features to LC-MS/MS peptide identifications

We calculated parts per million (ppm) difference between *m/z* values of peptides visualized by IMS and corresponding LC-MS/MS peptides (49). Using this ppm difference and several other metrics described previously (49), we determined probable identifications for 20 of the 24 differentially abundant peptides detected by IMS (Table S12). Specifically, IMS images were matched to probable LC-MS/MS peptide identifications using calculated parts per million difference of about 10 or less, the relative quantitative peptide abundance, and the peptide abundance patterns, as previously described (49).

### Pathway analysis

Using the four lists of proteins that were differentially abundant (increased or decreased in the corpus or antrum) when comparing WT-infected animals with high inflammation to *cag* mutant-infected animals, we converted Mongolian gerbil protein identifiers to official gene symbols. The official gene symbols were entered into the DAVID informatic resource as a gene list with *Meriones unguiculatus* (Mongolian gerbil) as the species. The resulting pathways were used for enrichment analyses. In some cases, one gene is mapped to multiple relevant pathways (in other words, there is overlap in the gene lists for some of the pathways). “Fold enrichment” refers to the percentage of altered genes that belong to the named pathway, divided by the corresponding percentage of total genes. “Count” refers to the number of altered genes that are mapped to the indicated pathway.

Figure 8 shows 12 representative pathways that exhibited significant differences when comparing WT-infected animals with high inflammation to *cag* mutant-infected animals. Additional pathways (not depicted in Fig. 8) for proteins with increased abundance in the antrum include glycolytic process, COPII-coated ER to Golgi transport vesicle, peptide catabolic process amyotrophic lateral sclerosis, spinocerebellar ataxia, ER exit site, COPII vesicle coat, COPI vesicle coat, intra-Golgi vesicle-mediated transport, SNAP receptor activity, SNARE interactions in vesicular transport, pertussis, NuRD complex, nucleocytoplasmic transport, structural constituent of nuclear pore, protein import into nucleus, and nuclear pore. Additional pathways (not depicted in Fig. 8) for proteins with increased abundance in the corpus include spinocerebellar ataxia, amyotrophic lateral sclerosis, DNA unwinding involved in DNA replication, MCM complex, and barbed-end actin filament capping.

## Data Availability

The mass spectrometry proteomics data have been deposited to the ProteomeXchange Consortium via the PRIDE partner repository with the data set identifier PXD060632.

## SUPPLEMENTAL MATERIAL

Supplemental figures iai.00595-24-s0001.pdf Fig. S1 and S2.

Supplemental tables iai.00595-24-s0002.xlsx Tables S1 to S13.

ASM does not own the copyrights to Supplemental Material that may be linked to, or accessed through, an article. The authors have granted ASM a non-exclusive, world-wide license to publish the Supplemental Material files. Please contact the corresponding author directly for reuse.
