# Supplementary material for: Helicobacter pylori CagA and Cag type IV secretion system activity have key roles in triggering gastric transcriptional and proteomic alterations
Source: Infect Immun. 2025 Mar 6;93(4):e00595-24. doi: 10.1128/iai.00595-24 (PMC11977315; doi:10.1128/iai.00595-24)
Supplement: Supplemental figures — Fig. S1 and S2. [file iai.00595-24-s0001.pdf]

Supplementary Figure 1

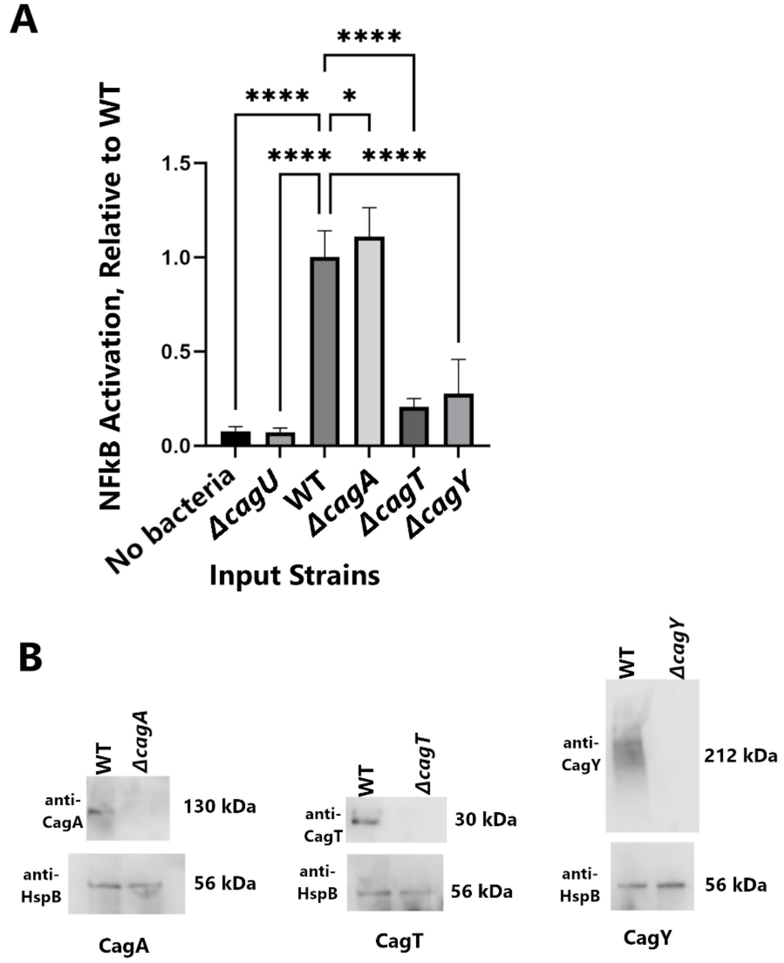

Supplementary Figure 1: Cag T4SS functionality in wild-type (WT) and *cag* mutant *H. pylori* strains. A) NF-κB activation by WT *H. pylori* 7.13 or *cag* mutant *H. pylori* strains. AGS NF-kB luciferase reporter cells were incubated with the indicated strains for 2.5 hours, and then relative luminescence was measured. All mutant strains were derived from *H. pylori* 7.13. A *ΔcagU* strain was tested as a Cag T4SS-negative control. "No bacteria" indicates uninfected cells. * indicates $p < 0.05$, **** indicates $p < 0.001$ (ANOVA with Dunnett's multiple test correction). B) Western blots of WT and mutant strains to detect CagA, CagT, and CagY, using rabbit polyclonal antisera. Anti-HspB was used as a loading control. Protein sizes are indicated to the right of the relevant proteins.

Supplementary Figure 2

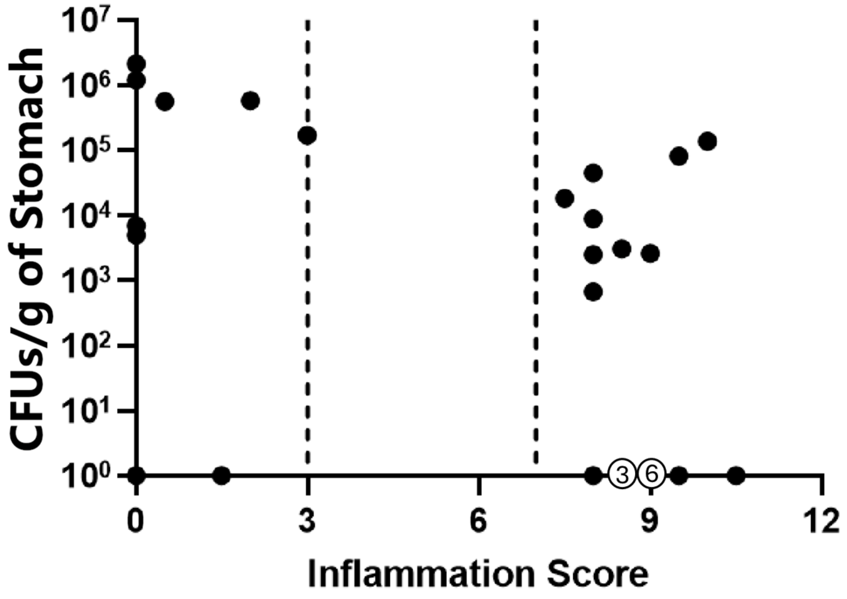

Supplementary Figure 2: Gastric inflammation and *H. pylori* colonization density in animals infected with a wild-type (WT) *H. pylori* strain. Vertical dashed lines indicate the inflammation score cutoffs for the “high inflammation” (>7) and “low inflammation” (<3) groups. Points on the graph may represent more than one animal in cases where the graphical coordinates are the same; this is indicated by open circles with the number of animals at that coordinate.
